# Predictors and Variability of Urinary Paraben Concentrations in Men and Women, Including before and during Pregnancy

**DOI:** 10.1289/ehp.1104614

**Published:** 2012-06-21

**Authors:** Kristen W. Smith, Joe M. Braun, Paige L. Williams, Shelley Ehrlich, Katharine F. Correia, Antonia M. Calafat, Xiaoyun Ye, Jennifer Ford, Myra Keller, John D. Meeker, Russ Hauser

**Affiliations:** 1 Department of Environmental Health, and; 2 Department of Biostatistics, Harvard School of Public Health, Boston, Massachusetts, USA; 3 Division of Reproductive Medicine, Department of Obstetrics, Gynecology, and Reproductive Biology, Brigham and Women’s Hospital, Boston, Massachusetts, USA; 4 National Center for Environmental Health, Centers for Disease Control and Prevention, Atlanta, Georgia, USA; 5 Department of Environmental Health Sciences, University of Michigan, Ann Arbor, Michigan, USA; 6 Vincent Memorial Obstetrics and Gynecology Service, Massachusetts General Hospital, Boston, Massachusetts, USA

**Keywords:** biomarker, exposure science, female, male, parabens, partners, predictors, pregnancy, variability

## Abstract

Background: Parabens are suspected endocrine disruptors and ubiquitous preservatives used in personal care products, pharmaceuticals, and foods. No studies have assessed the variability of parabens in women, including during pregnancy.

Objective: We evaluated predictors and variability of urinary paraben concentrations.

Methods: We measured urinary concentrations of methyl (MP), propyl (PP), and butyl paraben (BP) among couples from a fertility center. Mixed-effects regression models were fit to examine demographic predictors of paraben concentrations and to calculate intraclass correlation coefficients (ICCs).

Results: Between 2005 and 2010, we collected 2,721 spot urine samples from 245 men and 408 women. The median concentrations were 112 µg/L (MP), 24.2 µg/L (PP), and 0.70 µg/L (BP). Urinary MP and PP concentrations were 4.6 and 7.8 times higher in women than men, respectively, and concentrations of both MP and PP were 3.8 times higher in African Americans than Caucasians. MP and PP concentrations we CI re slightly more variable in women (ICC = 0.42, 0.43) than men (ICC = 0.54, 0.51), and were weakly correlated between partners (*r* = 0.27–0.32). Among 129 pregnant women, urinary paraben concentrations were 25–45% lower during pregnancy than before pregnancy, and MP and PP concentrations were more variable (ICCs of 0.38 and 0.36 compared with 0.46 and 0.44, respectively).

Conclusions: Urinary paraben concentrations were more variable in women compared with men, and during pregnancy compared with before pregnancy. However, results for this study population suggest that a single urine sample may reasonably represent an individual’s exposure over several months, and that a single sample collected during pregnancy may reasonably classify gestational exposure.

Parabens are a family of esters of *p*-hydroxybenzoic acid used as antimicrobial preservatives in multiple products including personal care products, pharmaceuticals, and foods (Andersen 2008; National Toxicology Program 2005; Orth 1980), and several parabens are often used in combination (Andersen 2008; Soni et al. 2005). Exposure may occur through ingestion, inhalation, or dermal absorption. Following excretion, the parent compounds can be measured in urine and have been shown to be valid biomarkers of exposure (Ye et al. 2006a). For example, measurable levels of several parabens have been found in the general U.S. population (Calafat et al. 2010; Ye et al. 2006a). Methyl paraben (MP) and propyl paraben (PP) have been detected in the urine of > 92% of a representative sample of the U.S. population participating in the 2005–2006 National Health and Nutrition Examination Survey (NHANES), whereas butyl paraben (BP) was detected in 47% of the urine samples tested (Calafat et al. 2010).

Parabens are suspected endocrine disruptors that are weakly estrogenic (Golden et al. 2005; Routledge et al. 1998; Soni et al. 2005) and antiandrogenic (Darbre and Harvey 2008), although their level of toxicity is thought to be low (Golden et al. 2005; Soni et al. 2005). Based on limited toxicological data, the U.S. Food and Drug Administration (FDA) designated MP and PP as generally recognized as safe (FDA 2006). Limited animal studies have reported adverse effects of some parabens on male (Kang et al. 2002; Oishi 2001, 2002a, 2002b) and female (Kang et al. 2002; Taxvig et al. 2008; Vo et al. 2010) reproductive and endocrine function, although others have not (Hoberman et al. 2008; Shaw and deCatanzaro 2009). Human data on the reproductive health effects of paraben exposure are limited, with one study reporting a positive association of MP and BP urinary concentrations with sperm DNA damage (Meeker et al. 2010). As far as we know, no human studies have reported evidence of female reproductive health effects or developmental effects associated with *in utero* exposure. One previous study examined the temporal variability of paraben exposure in men (Meeker et al. 2010). However, we are unaware of any studies that have examined variability in women before or during pregnancy. Understanding urinary paraben variability is important because parabens are excreted within hours after exposure (Janjua et al. 2008), whereas health effects are likely related to recurrent exposures that take place over time. Also, because the fetus may be especially vulnerable to *in utero* exposures, it is important to assess gestational exposure and its variability during pregnancy.

Given the high detection frequency of some parabens in the U.S. population (Calafat et al. 2010), the objectives of this study were to evaluate the variability and demographic predictors of urinary paraben concentrations in 653 adult men and women, some of whom were partners. Specifically, our objectives were to *a*) characterize urinary paraben concentrations among study participants; *b*) evaluate differences in urinary paraben concentrations by demographic factors (age, sex, and race); and *c*) evaluate the variability of urinary paraben concentrations among men and women, and variability before and during pregnancy in a subset of women who became pregnant during study follow-up.

## Methods

*Subjects.* Participants were male and female patients (some of whom were couples) from the Fertility Center at the Massachusetts General Hospital (MGH) who were recruited into a prospective cohort study on environmental risk factors for reproductive health and contributed at least one urine sample for measuring environmental chemicals, including parabens. All patients > 18 years of age seeking infertility evaluation or treatment at the MGH Fertility Center were eligible to participate, and approximately 60% consented. We recruited participants between December 2004 and October 2010 and followed them from study entry until discontinuation of fertility treatment, a live birth, or loss to follow-up. The human studies institutional review boards of the MGH, Harvard School of Public Health (HSPH), and the Centers for Disease Control and Prevention (CDC) approved the study. Participants signed an informed consent after the study procedures were explained by a research nurse and all questions were answered.

*Urine sample collection.* We collected a spot urine sample from study participants at the time of recruitment, at subsequent visits during infertility treatment cycles, and, if applicable, during pregnancy. Convenience (spot) samples were collected between August 2005 and November 2010. Urine samples collected before August 2005 were not analyzed for parabens because these chemicals were added to the study protocol after that date. Urine was collected in a sterile polypropylene cup. After specific gravity (SG) was measured using a handheld refractometer (National Instrument Company, Inc., Baltimore, MD), the urine was divided into aliquots and frozen at –80°C. Samples were shipped on dry ice overnight to the CDC (Atlanta, GA), where concentrations of total (free + conjugated) MP, PP, and BP were measured using on-line solid phase extraction-high performance liquid chromatography–isotope dilution tandem mass spectrometry as previously reported (Ye et al. 2006b). The limits of detection (LOD) were 1.0 μg/L for MP and 0.2 μg/L for PP and BP.

*Demographic predictors of paraben concentrations.* Information on demographic factors of interest, collected through nurse-administered and take-home questionnaires, included sex, race, and age, which were previously shown to be associated with urinary paraben concentrations in the general population (Calafat et al. 2010). Race was categorized as Caucasian, African American, Asian, and other. We also examined urinary concentrations of parabens according to body mass index (BMI) categorized as underweight (< 18.5 kg/m^2^), normal (18.5–24.9 kg/m^2^), overweight (25–29.9 kg/m^2^), and obese (≥ 30 kg/m^2^). Weight and height were measured by a research nurse at study entry.

*Statistical analysis.* We evaluated demographic characteristics of male and female study participants (means and percentages). We report the distribution of urinary paraben concentrations for all individual samples, and also report the distribution of within-person geometric mean (GM) values because the number of urine samples from each participant varied and within-person concentrations were log-normally distributed. These data are uncorrected for SG to allow comparison with other studies.

We replaced paraben concentrations less than the LOD with LOD divided by the square root of 2 (Hornung and Reed 1990). We calculated the Spearman correlation between the different parabens. We corrected the urinary paraben concentrations for SG using a modification of a previously described formula: *Pc* = *P*[(1.016 – 1)/*SG* – 1], where *Pc* is the *SG*-corrected paraben concentration (micrograms per liter), 1.016 is the mean *SG* for the samples examined, and *P* is the measured paraben concentration (micrograms per liter) (Duty et al. 2005). Natural log-transformed SG-corrected paraben concentrations were used as the outcome in all statistical models. We excluded BP from further statistical analyses including both males and females due to a low detection frequency (65% detected).

We fit linear mixed-effects models to estimate associations of urinary MP and PP concentrations (micrograms per liter urine) with age, sex, race, and BMI, with each paraben modeled separately. We included a random effect for subject in the models to account for correlation among repeat samples collected on the same individual over time. Sex, race, and BMI were included as fixed effects, whereas age at urine collection was included as a time varying factor. Using step-wise backward elimination, we retained covariates with a *p*-value < 0.1. Final models included sex, race, and BMI. The parameter estimates were exponentiated to estimate the difference in paraben concentrations relative to the reference category of each predictor variable.

To determine whether couples have similar patterns of paraben exposure, we calculated Spearman correlation coefficients for within-person GM paraben concentrations between partners, as well as for paraben concentrations between partners with urine samples that were collected on the same day (time matched).

To examine the reproducibility of urinary MP and PP concentrations, we calculated intraclass correlation coefficients (ICCs) using SAS PROC MIXED (SAS Institute Inc., Cary, NC) with a random effect for subject for participants who provided at least two urine samples. The ICC is calculated as the ratio of between-person variability to total variability (total variability = between-person + within-person variability). ICCs closer to zero indicate less reproducibility (large within-person variability) and ICCs closer to one indicate higher reproducibility (low within-person variability). Rosner (1995) defined an ICC < 0.4 as indicating poor reproducibility, an ICC between 0.4 and < 0.75 as indicating fair to good reproducibility, and an ICC ≥ 0.75 as indicating excellent reproducibility.

*Subset analysis of pregnant women.* To compare the variability of urinary paraben concentrations before and during pregnancy, we evaluated a subset of women who became pregnant during follow-up and had provided at least two prepregnancy and at least two pregnancy spot urine samples. An intrauterine pregnancy was defined by the presence of a fetal heart beat detected by transvaginal ultrasound. We assigned urine samples to a trimester based on the following definition: first trimester: 0–13.9 weeks gestation; second trimester: 14.0–28.0 weeks; and third trimester: ≥ 28.1 weeks. We assigned the gestational week of the urine sample collection using the estimated date of conception, which was defined as the expected date of delivery minus 266 days. We estimated the delivery date using three dating methods (in order of preference if more than one was available): *a*) oocyte retrieval date as recorded from medical records; *b*) crown–rump length as measured by first trimester ultrasound; or *c*) women’s reported date of last menstrual period.

We calculated the within-woman GM for prepregnancy and pregnancy urinary paraben concentrations and report the median and 25th–75th percentiles [interquartile range (IQR)]. We also report urinary paraben concentrations (median and IQR) for samples collected in each trimester of pregnancy. We estimated the Spearman correlation between the GM paraben concentrations before and during pregnancy.

We fit linear mixed-effects models with a random effect for subject to estimate the change in urinary paraben concentrations before and during pregnancy. First, we used pregnancy status (before vs. during) to estimate the difference in urinary paraben concentrations during pregnancy as compared with before pregnancy. Second, restricting to urine samples collected during pregnancy, we estimated the change in urinary paraben concentrations over continuous time in weeks since conception. We exponentiated the parameter estimates to estimate the percent change in the paraben concentration per week since conception. We evaluated the reproducibility of urinary MP, PP, and BP concentrations before and during pregnancy by calculating the ICCs for samples collected during each time period.

Finally, we conducted a classification analysis (Hauser et al. 2004; Mahalingaiah et al. 2008) using the GM of the two or three urine samples collected during pregnancy as the gold-standard exposure measure. We divided this GM summary exposure measure into tertiles, as well as each trimester-specific concentration (using trimester-specific tertile cut points). We calculated the sensitivity, specificity, and positive predictive value (PPV) of each trimester-specific paraben concentration to correctly classify a woman into the highest exposure tertile (based on the gold standard). To minimize bias in this analysis we excluded women with all of their urine samples collected in the same trimester (*n* = 3 women). Among women with two samples collected in the same trimester (who also had one other sample collected in another trimester) we included only the first of the two samples collected in the same trimester (*n* = 4 women). In a second analysis restricted to women with one urine sample collected in each trimester, we counted the number of women who remained in the same exposure tertile over the course of pregnancy (Braun et al. 2012). Each woman could have either zero, two, or all three urine samples remaining in the same tertile during pregnancy. We conducted all statistical analyses using SAS version 9.2 (SAS Institute Inc.). We made no adjustment for multiple comparisons.

## Results

We measured urinary paraben concentrations in 2,721 spot samples collected from 653 male and female participants. Participants contributed 4 urine samples on average (median = 3), ranging from 1 (141 participants) to 19 (2 participants). A total of 408 women contributed 2,128 samples, and 245 men contributed 593 samples. Seven urine samples had missing SG values, and three urine samples had implausible SG values (> 1.04) that were set to missing. There were 226 couples, of whom 197 couples provided at least one pair of time-matched samples (both partners’ urine samples collected on the same day). On average, each couple had 2 pairs of samples (median = 2), and there were 419 pairs of time-matched samples ranging from 1 (82 couples) to 7 pairs (1 couple). Due to missing SG data from one subject, one couple was excluded from this analysis (final *n* = 225).

Participating men and women were primarily Caucasian, ranged in age from 21 to 57 years (mean: 36 years; SD: 4.8 years), had a BMI in the normal to overweight range, and were highly educated (Table 1).

**Table 1:** Demographic characteristics of study participants.

Characteristic	All subjects (*n* = 653)	Females (*n* = 408)	Males (*n* = 245)
Age at enrollment (years) [mean ± SD (range)]	36.0 ± 4.8 (20.9–56.8)	35.7 ± 4.2 (20.9–46.7)	36.5 ± 5.5 (23.9–56.8)
Race [n (%)]
Caucasian	552 (85)	339 (83)	213 (87)
African American	27 (4)	18 (4)	9 (4)
Asian	43 (7)	28 (7)	15 (6)
Other	31 (5)	23 (6)	8 (3)
BMI [mean ± SD (range)]a	25.9 ± 4.9 (16.5–49.0)	24.9 ± 5.0 (16.5–49.0)	27.5 ± 4.3 (19.3–47.9)
BMI at enrollment (kg/m2) [n (%)]a
Underweight (< 18.5)	6 (1)	6 (2)	0 (0)
Normal (18.5–24.9)	318 (49)	249 (61)	69 (28)
Overweight (25–29.9)	210 (32)	89 (22)	121 (50)
Obese (≥ 30)	116 (18)	62 (15)	54 (22)
Education [n (%)]b
Did not graduate from college	62 (13)	31 (10)	31 (18)
College graduate	164 (34)	107 (34)	57 (32)
Graduate degree	263 (54)	174 (56)	89 (50)
an = 650. bn = 489.						

MP and PP were detected in over 90% of samples collected from men and women, whereas BP was detected in 74% of samples from women and 36% of samples from men (Table 2). The median paraben concentrations using both the individual samples and the within-subject GM increased in the following order: MP > PP > BP, and were higher in women compared with men, and higher among African Americans compared with Caucasians (Table 2). There was a strong correlation between MP and PP (Spearman *r* = 0.86) and a moderate correlation for MP and BP (Spearman *r* = 0.49) and PP and BP (Spearman *r* = 0.47).

**Table 2:** Distribution of urinary paraben concentrations (µg/L) (*n* = 2,721 samples) among 653 study participants.^a^

Analyte	Individual samples								Within-person GM^b^					
	*n*	Percent detect^c^	GM	Minimum	25th percentile	50th percentile	75th percentile	Maximum	*n*	Minimum	25th percentile	50th percentile	75th percentile	Maximum
MP
All subjects	2,721	99.7	100	< LOD	31.3	112	354	23,200	653	< LOD	30.4	82.2	236	7,110
Sex
Female	2,128	99.9	137	< LOD	48.8	155	422	15,100	408	2.56	55.1	149	299	7,110
Male	593	99.3	33.0	< LOD	10.0	29.0	96.7	23,200	245	< LOD	12.8	31.2	81.0	2,880
Race
Caucasian	2,320	99.7	97.1	< LOD	29.9	104	332	23,200	552	< LOD	28.0	75.3	219	7,110
African American	87	100	343	8.10	158	362	868	4,730	27	57.6	158	340	907	3,880
Asian	164	100	92.2	1.20	28.5	104	346	1,860	43	4.57	25.1	91.0	241	1,073
Other	150	100	92.3	1.80	31.1	96.4	319	3,330	31	6.00	37.9	97.0	204	1,308
PP
All subjects	2,721	96.5	17.9	< LOD	4.00	24.2	90.2	2,870	653	< LOD	3.49	15.4	53.1	2,510
Sex
Female	2,128	98.3	27.5	< LOD	7.90	34.3	118	2,870	408	< LOD	9.75	28.1	78.9	2,510
Male	593	90.2	3.82	< LOD	0.80	3.10	16.8	1,170	245	< LOD	0.84	3.30	15.2	667
Race
Caucasian	2,320	96.8	17.7	< LOD	4.00	23.6	87.0	2,550	552	< LOD	3.24	14.6	47.8	2,510
African American	87	100	63.0	1.30	22.4	88.4	198	1,170	27	2.00	15.9	95.6	177	318
Asian	164	95.7	16.3	< LOD	3.85	22.2	93.2	909	43	0.24	3.00	18.9	40.6	370
Other	150	90.7	10.8	< LOD	1.80	13.2	84.3	2,870	31	< LOD	3.60	10.9	52.8	467
BP
All subjects	2,721	65.4	1.08	< LOD	< LOD	0.70	5.40	998	653	< LOD	< LOD	0.59	2.80	208
Sex
Female	2,128	73.6	1.48	< LOD	< LOD	1.20	7.65	595	408	< LOD	0.35	1.30	4.47	128
Male	593	35.9	0.35	< LOD	< LOD	< LOD	0.50	998	245	< LOD	< LOD	< LOD	0.70	208
Race
Caucasian	2,320	65.8	1.11	< LOD	< LOD	0.70	5.80	998	552	< LOD	< LOD	0.59	2.80	208
African American	87	77.0	1.34	< LOD	0.20	1.10	6.10	93.8	27	< LOD	< LOD	0.96	4.09	27.3
Asian	164	57.9	0.88	< LOD	< LOD	0.35	3.75	194	43	< LOD	< LOD	0.44	2.19	95.1
Other	150	61.3	0.77	< LOD	< LOD	0.50	2.80	112	31	< LOD	< LOD	0.44	2.19	95.1
aValues were not corrected for SG to facilitate comparison with other studies. bThe within-person GM was used as a summary exposure measure for each subject. cLOD: MP = 1 µg/L; PP = 0.2 µg/L; BP = 0.2 µg/L.																		

In multivariable regression models, sex, race, and BMI were included as predictors of urinary concentrations of MP and PP. Concentrations of MP and PP were 4.55 [95% confidence interval (CI): 3.73, 5.56) and 7.81 (95% CI: 6.00, 10.2) times higher in women compared with men, and were 3.84 (95% CI: 2.40, 6.13) and 3.80 (95% CI: 2.05, 7.06) times higher in African Americans compared with Caucasians (Table 3). Concentrations of MP and PP were 0.79 (95% CI: 0.61, 1.01) and 0.77 (95% CI: 0.55, 1.07) times lower in obese participants (BMI ≥ 30) compared with participants with a normal BMI (18.5–24.9) (Table 3).

**Table 3:** Relative change (95% CI) in urinary paraben concentrations as a function of demographic and anthropometric predictors from a multivariate regression model.

Parameters	*n* Subjects (*n* samples)^a^	MP	PP
		Relative change (95% CI)^b^	Relative change (95% CI)^b^
Sex
Female	405 (2,104)	4.55 (3.73, 5.56)	7.81 (6.00, 10.2)
Male	243 (589)	1 (Reference)	1 (Reference)
Race
African American	27 (87)	3.84 (2.40, 6.13)	3.80 (2.05, 7.06)
Asian	43 (163)	0.99 (0.69, 1.43)	0.93 (0.57, 1.49)
Other	31 (149)	1.00 (0.66, 1.51)	0.74 (0.43, 1.28)
Caucasian	547 (2,294)	1 (Reference)	1 (Reference)
BMI (kg/m2)
Obese (≥ 30)	116 (423)	0.79 (0.61, 1.01)	0.77 (0.55, 1.07)
Overweight (25–29.9)	210 (778)	0.95 (0.77, 1.16)	0.91 (0.69, 1.19)
Normal (18.5–24.9)	316 (1,457)	1 (Reference)	1 (Reference)
Underweight (< 18.5)	6 (35)	1.42 (0.57, 3.57)	1.46 (0.43, 4.92)
an = 648 subjects and 2,693 urine samples (reduced sample size due to missing BMI and SG values). bExponentiated adjusted parameter estimates from multivariate regression models (including sex, race, and BMI) are presented due to natural log transformation of the outcome and can be interpreted as a relative (times) change from the reference category of the predictor variable; each paraben is modeled separately.						

Among 225 couples, correlations of MP (*r* = 0.27) and PP (*r* = 0.32) between partners were relatively weak. Among 196 couples with urine samples collected on the same day the correlations were similar for MP (*r* = 0.28) and PP (*r* = 0.33).

A total of 511 study participants (346 females and 165 males) provided more than one urine sample (2,059 female and 513 male samples) and contributed to the ICC calculations. The time between collection of the first and last urine sample for each study participant ranged from 2 to 1,273 days with a mean (± SD) of 271.5 ± 232.6 days. Urinary paraben concentrations exhibited slightly higher within-person variability among female (ICC: MP = 0.42, PP = 0.43) than male study participants (ICC: MP = 0.54, PP = 0.51). Among women who became pregnant during study follow-up, the prepregnancy ICCs were similar to women overall (ICC: MP = 0.46, PP = 0.44).

During study follow-up, 129 women became pregnant, resulting in 124 live births and 3 stillbirths (absence of fetal heart beat after 20 weeks gestation); 2 women were lost to follow-up. The 129 women provided 912 urine samples: 575 samples before pregnancy (2–14 samples/woman) and 337 samples during pregnancy (2–3 samples/woman). On average, first-, second-, and third-trimester urine samples were collected at gestational weeks 5.8 (range: 3–13.6), 20.6 (range: 14.9–27.0), and 33.5 (range: 28.4–37.6), respectively. Women generally provided urine samples in different trimesters, although 7 women provided two urine samples during the first trimester. One woman was excluded from the analysis due to missing SG data, and another because of a missing expected date of delivery. Two prepregnancy urine samples were excluded from the analysis due to missing SG data. If a patient re-enrolled in the study and became pregnant a second time, only the first pregnancy was included in this analysis (*n* = 3).

The detection frequencies for MP and PP were similar before and during pregnancy (MP: both 100%; PP: prepregnancy: 98%, pregnancy: 99%), but more samples had detectable BP before pregnancy (79%) than during pregnancy (70%). Within-person GM urinary MP, PP, and BP concentrations were lower during pregnancy compared with before pregnancy (Table 4), with moderate correlations before and during pregnancy (Spearman *r* = 0.55, 0.56, 0.55, respectively). Before pregnancy, the ICCs were 0.46 (MP), 0.44 (PP), and 0.49 (BP), but were lower or similar during pregnancy (MP = 0.38, PP = 0.36, BP = 0.48).

**Table 4:** Relationship between within-woman geometric mean prepregnancy and pregnancy urinary paraben concentrations among 129 women.^a^

Analyte	Uncorrected median (IQR)		SG-corrected median (IQR)		Relative change (95% CI)^b^
	Prepregnancy	Pregnancy	Prepregnancy	Pregnancy	
MP (µg/L)	162 (63.6, 334)	135 (51.3, 287)	201 (103, 400)	185 (69.3, 348)	0.75 (0.64, 0.88)
PP (µg/L)	36.1 (13.6, 78.2)	22.8 (7.33, 75.2)	46.4 (20.1, 98.3)	36.3 (10.3, 89.9)	0.68 (0.56, 0.82)
BP (µg/L)	2.39 (0.45, 5.45)	0.88 (0.25, 2.88)	2.96 (0.73, 8.36)	1.23 (0.42, 4.03)	0.55 (0.45, 0.67)
aThe median of the within-woman GM paraben urinary concentrations are presented (n = 129 women contributing 575 urine samples prepregnancy; n = 129 women contributing 337 urine samples during pregnancy). bResults from a mixed-effects linear regression model evaluating the change in SG-corrected MP, PP, and BP during pregnancy compared with before pregnancy (no additional covariates were included in the model). The relative change from the reference category (prepregnancy) is presented; n = 912 urine samples were included in the model: 575 prepregnancy and 337 during pregnancy.										

Median urinary paraben concentrations (with and without SG correction) were lower for the second and third trimesters than the first trimester [see Supplemental Material, Table S1 (http://dx.doi.org/10.1289/ehp.1104614)]. Estimates from mixed effects regression models restricted to samples collected during pregnancy suggested a decrease in urinary paraben concentrations with each additional week of pregnancy for MP (–0.9%; 95% CI: –2.0%, 0.3%) and PP (–1.2%; 95% CI: –2.7%, 0.2%), but not for BP (–0.2%; 95% CI: –1.5%, 1.1%).

Overall, among 126 women with 2–3 urine samples collected in separate trimesters, the first- and second-trimester urinary MP concentrations, second-trimester PP concentrations, and third-trimester BP concentrations appeared to be the most accurate for classifying gestational exposure based on the sensitivity, specificity and PPV for the probability that the overall GM would be in the highest tertile given a trimester-specific urine sample in the highest tertile (Table 5). Among women with a urine sample from each trimester of pregnancy (*n* = 75), at least 85% remained in the same urinary paraben exposure tertile for at least two trimesters [see Supplemental Material, Table S2 and, for urinary paraben exposure tertile cut points, Table S3 (http://dx.doi.org/10.1289/ehp.1104614)].

**Table 5:** Sensitivity, specificity, and positive predictive value of a trimester-specific urinary paraben concentration to predict the highest tertile of the GM gestational urinary paraben concentration.^a^

Trimester^*b*^	*n*^*b*^	MP			PP			BP		
		Sensitivity	Specificity	PPV	Sensitivity	Specificity	PPV	Sensitivity	Specificity	PPV
1st	120	0.73	0.86	0.73	0.63	0.82	0.65	0.70	0.85	0.70
2nd	121	0.73	0.86	0.73	0.73	0.86	0.73	0.73	0.86	0.73
3rd	86	0.64	0.79	0.55	0.63	0.80	0.59	0.83	0.93	0.86
aThe gold standard is the GM gestational urinary paraben concentration (2–3 urine samples per subject). Classification probabilities are based on the highest versus two lowest tertiles of gestational or trimester-specific urinary paraben concentrations. bThere is a maximum of one urine sample included per subject per trimester; n = 126 women included in analysis.																				

## Discussion

Similar to a previous study evaluating paraben exposure in the general U.S. population (Calafat et al. 2010), concentrations of parabens in this study were highest for MP followed by PP and then BP. MP and PP were highly correlated in our study, suggesting a common source of exposure, whereas their correlation with BP was lower, suggesting fewer common exposure sources. MP and PP are the most commonly used parabens (Soni et al. 2005), and are often used in products such as foods or cosmetics in combination (Soni et al. 2005), whereas BP is less widely used. Ethyl-paraben (EP) was not measured in this study because of relatively low detection rates compared with other parabens in the U.S. population (Calafat et al. 2010).

Urinary MP and PP concentrations were more than four times higher in women compared with men, and more than three times higher among African Americans compared with Caucasians. These results were similar to a previous study among the general U.S. population (Calafat et al. 2010) that also found that females had significantly higher concentrations of MP and PP compared with males, and that non-Hispanic blacks had significantly higher concentrations of MP and PP compared with non-Hispanic whites. As noted by Calafat et al. (2010) this relationship could be attributable to product use or pharmacokinetic differences between males and females and between African Americans and Caucasians. The low proportion of non-Caucasians in our sample limited our ability to precisely estimate race-specific values. MP and PP concentrations were lower among obese participants than participants with a normal BMI. This suggests that pharmacokinetic differences may contribute to variation in urinary paraben concentrations or that individuals with a higher BMI have different exposure profiles with regard to personal care products, medications, or food. These results are consistent with a previous report of an inverse relationship between BMI and urinary parabens in males (Meeker et al. 2010). The 82 men from the previous report were recruited from the same fertility center, but there was no overlap with the current study sample.

Correlations of MP and PP concentrations between partners were low, including correlations between paired urine samples collected on the same day. This may reflect differences in diet and the use of different personal care products and medications.

One previous study reported data on temporal variability of urinary parabens for 82 men (Meeker et al. 2010), but we are not aware of any previous studies reporting the variability of urinary paraben concentrations in women. Among men and women there was moderate within-person variability in MP and PP, with slightly more variability among women compared with men. Potential explanations for the higher variability in women may be the collection of some urine samples during pregnancy, and also possibly changes in personal care product use over time.

Among women who became pregnant during follow-up, paraben concentrations were lower during pregnancy than before pregnancy. There also was higher within-woman variability of MP and PP urinary concentrations during pregnancy than before pregnancy, and a suggestive decrease in paraben concentrations with each additional week of pregnancy. Although understudied, it is possible that parabens may affect fetal growth. If this were the case, gestational age estimated using crown–rump length could be less accurate among women with higher urinary paraben concentrations, potentially biasing estimates of the change in paraben concentrations with each additional week of pregnancy.

Differences in urinary paraben concentrations before and during pregnancy, and increases in within-woman variability for MP and PP, could be attributable to changes in the use of personal care products and medications or food consumption during pregnancy. Although women participating in this study may have chosen to change their habits once they became pregnant, results from this study population may not be generalizable to pregnant women overall because our participants were primarily Caucasian, older, and more highly educated than the general population of pregnant women. However, differences also could reflect physiological changes that may affect absorption, distribution, metabolism, and/or excretion of parabens during pregnancy (Woodruff et al. 2011).

Based on the fair reproducibility of urinary paraben concentrations among men and women (ICCs for MP and PP ranged from 0.42–0.54), a single urine sample may reasonably represent an individual’s exposure over several months. Due to the design of the parent research project, the time period over which samples were collected varied widely by study participant and ranged from days to a few years. Although it would be ideal to collect multiple urine samples to evaluate gestational exposure given the low to moderate ICCs (MP = 0.38, PP = 0.36, BP = 0.48), the accuracy of trimester-specific paraben concentrations in classifying gestational exposure into the highest tertile (Table 5), as well as the high proportion of women remaining in the same exposure tertile for at least two trimesters of pregnancy (> 85%), indicate that a urine sample collected anytime during pregnancy may reasonably classify gestational exposure. However, a limitation of the classification analysis is that the gold-standard exposure measure was derived from only two or three trimester-specific concentrations for each woman.

Although we collected multiple urine samples from women and their partners, it is unlikely that we captured all of the urinary paraben variability. Similar to other nonpersistent compounds for which exposure is episodic in nature (Mahalingaiah et al. 2008; Preau et al. 2010), paraben concentrations may fluctuate throughout the day, adding additional within-person variability that could not be accounted for in this study. Also, the generalizability of our findings may be limited because our study population was primarily Caucasian and highly educated.

These findings suggest that urinary paraben concentrations differ according to demographic factors and pregnancy status in our study population. In addition, our results suggest that a single urine sample may reasonably represent an individual’s exposure over several months, and that a single urine sample collected during pregnancy may reasonably classify gestational exposure in this group of participants.

## Supplemental Material

(33 KB) PDF
