# Supplementary material for: Predictors and Variability of Urinary Paraben Concentrations in Men and Women, Including before and during Pregnancy
Source: Environ Health Perspect. 2012 Jun 21;120(11):1538–43. doi: 10.1289/ehp.1104614 (PMC3556607; doi:10.1289/ehp.1104614)
Supplement: (33 KB) PDF [file ehp.1104614.s001.pdf]

Supplemental Material

Predictors and Variability of Urinary Paraben Concentrations in Men and Women, Including before and during Pregnancy

Kristen W. Smith, Joe M. Braun, Paige L. Williams, Shelley Ehrlich, Katharine F. Correia, Antonia M. Calafat, Xiaoyun Ye, Jennifer Ford, Myra Keller, John D. Meeker, Russ Hauser

Table of contents:

Supplemental Material, Table S1. Urinary paraben concentrations in samples collected during each trimester of pregnancy…………………………………………………………...…….Page 2

Supplemental Material, Table S2. Number (percent) of women with trimester specific urinary paraben samples remaining in the same exposure tertile during pregnancy……………….…..Page 3

Supplemental Material, Table S3. Trimester specific urinary paraben tertile cutpoints…….Page 4

Supplemental Material, Table S1. Urinary paraben concentrations in samples collected during each trimester of pregnancy[a]

	% Detect	Median (IQR)	
		Uncorrected	SG-corrected
Methyl Paraben (µg/L)			
1st Trimester	100%	175 (37.8, 545)	209 (54.6, 567)
2nd Trimester	100%	130 (41.7, 275)	153 (56.8, 336)
3rd Trimester	98.8%	120 (37.2, 278)	175 (63.7, 341)
Propyl Paraben (µg/L)			
1st Trimester	99.2%	46.6 (6.60, 147)	61.4 (9.54, 158)
2nd Trimester	97.5%	25.7 (5.20, 92.4)	33.6 (7.04, 119)
3rd Trimester	98.8%	28.1 (5.60, 74.5)	40.1 (8.09, 103)
Butyl Paraben (µg/L)			
1st Trimester	71.5%	0.70 (0.14, 5.00)	1.13 (0.36, 5.06)
2nd Trimester	69.4%	0.70 (0.14, 4.50)	0.85 (0.25, 5.44)
3rd Trimester	67.4%	0.60 (0.14, 3.90)	0.82 (0.32, 6.24)

Abbreviation: IQR = Interquartile Range

[a]Trimester 1: N=123 subjects (130 urine samples); Trimester 2: N=121 subjects (121 urine samples); Trimester 3: N=86 subjects (86 urine samples)

Supplemental Material, Table S2. Number (percent) of women with trimester specific urinary paraben samples remaining in the same exposure tertile during pregnancy[a]

Number of samples in same exposure tertile[b]	MP	PP	BP
Zero	8 (11%)	9 (12%)	11 (15%)
Two	49 (65%)	49 (65%)	46 (61%)
All	18 (24%)	17 (23%)	18 (24%)

[a]Among women with one urine sample from each trimester of pregnancy (N=75)

[b]Zero = Each of the three urine samples was categorized in a different exposure tertile throughout pregnancy; Two = Two of the three urine samples remained in the same exposure tertile during pregnancy; All = Each of the three urine samples remained in the same exposure tertile throughout pregnancy

Supplemental Material, Table S3. Trimester specific urinary paraben tertile cutpoints[a]

	33rd %tile	67th %tile
Methyl Paraben (µg/L)		
1st Trimester	69.0	358
2nd Trimester	81.6	224
3rd Trimester	100	267
Propyl Paraben (µg/L)		
1st Trimester	11.8	139
2nd Trimester	10.1	76.4
3rd Trimester	10.7	68.9
Butyl Paraben (µg/L)		
1st Trimester	0.45	3.00
2nd Trimester	0.36	2.74
3rd Trimester	0.53	2.51

Abbreviation: %tile = percentile

[a]Trimester 1: N=120 urine samples from 120 women; Trimester 2: N=121 urine samples from 121 women; Trimester 3: N=86 urine samples from 86 women; N=126 total women included
